# Characterization of Goat Meat Quality Under Different Forage Feeding Regimes

**DOI:** 10.3390/ani16142246

**Published:** 2026-07-20

**Authors:** Kyu-Min Kang, Jin-Ki Park, Kwanghyun Cho, Joon Mo Yeo, Jeong Sung Jung, Hyun-Jung Park, Won-Young Lee

**Affiliations:** 1 Department of Animal Resources Science, Kongju National University, Yesan 32439, Republic of Korea; rbals15@naver.com; 2 Department of Livestock, Korea National University of Agriculture and Fisheries, Jeonju-si 54874, Republic of Korea; parkjk@korea.kr (J.-K.P.); ckh1219@korea.kr (K.C.); yeoj@korea.kr (J.M.Y.); 3 Forage Production System Division, Department of Animal Resources Development, National Institute of Animal Science, Cheonan 31000, Republic of Korea; jjs3873@korea.kr; 4 Department of Biotechnology, College of Biomedical & Health Science, Konkuk University, Chungju 27475, Republic of Korea

**Keywords:** goat, Bermuda grass, perennial ryegrass, meat quality

## Abstract

In this study, we evaluated the effects of Bermuda grass and perennial ryegrass feeding on the proximate composition, shear force, fatty acid profile, and flavor characteristics of goat meat (rectus femoris). Thirty castrated Korean native black × Boer crossbred goats (10 months old) were divided into two groups (*n* = 15 per group) and fed forage-based diets consisting of 35% forage (either Bermuda grass or perennial ryegrass) and 65% commercial concentrate for 4 months. The Bermuda grass group showed a significantly higher fat content and lower shear force (*p* < 0.05), along with higher relative proportions of linoleic and α-linolenic acids. Aroma analysis revealed higher methanethiol and 2-methylheptanal levels in the Bermuda grass group, whereas taste profiling revealed a stronger umami flavor and lower sourness and saltiness. Overall, Bermuda grass feeding may improve selected meat quality characteristics of goat meat without adversely affecting growth performance. However, its nutritional implications should be interpreted cautiously because of the lower UFA/SFA ratio observed in the present study.

## Simple Summary

The type of forage fed to goats can influence the nutritional quality and eating characteristics of their meat. However, information comparing different forage sources is still limited. In this study, goats were fed either Bermuda grass or perennial ryegrass during the fattening period, and their growth performance, meat quality, fatty acid composition, aroma, and taste characteristics were evaluated. Although growth performance was similar between the two groups, differences were observed in the nutritional and flavor-related properties of the meat. Meat from goats fed perennial ryegrass contained higher proportions of some beneficial fatty acids, whereas Bermuda grass was associated with more favorable fatty acid ratios and differences in aroma- and taste-related instrumental measurements. These findings suggest that forage type can influence the nutritional and quality characteristics of goat meat without affecting animal growth. The results provide useful information for livestock producers seeking feeding strategies to improve meat quality and offer consumers a better understanding of how animal diets may influence the characteristics of goat meat.

## 1. Introduction

As global warming continues, abnormal climatic events are being increasingly observed worldwide. Northeast Asia, in particular, has been experiencing a shift from a temperate monsoon climate to a subtropical climate, accompanied by an increased frequency of extreme events such as floods and droughts [1]. These environmental changes negatively affect the chemical, physical, and biological properties of soils, thereby hindering the growth of forage crops and reducing their nutritional quality, ultimately impacting livestock productivity [2]. In response, the selection and cultivation of forage species that are well adapted to changing climatic conditions have become increasingly important.

Warm-season forage grasses such as Bermuda grass, Bahia grass, Kikuyu grass, and Rhodes grass are well suited for cultivation in subtropical climates due to their high resistance to environmental stresses and strong growth characteristics [3]. Among these, Bermuda grass is characterized by rapid propagation through seeds, stolons, and rhizomes, as well as strong tolerance to high temperatures and drought [4]. In addition to their agronomic advantages, these forages can influence rumen fermentation processes, thereby affecting nutrient utilization and livestock productivity [5].

When ruminants consume forage, the digestibility and chemical composition of the diet influence rumen microbial activity and fermentation patterns, which in turn regulate energy metabolism and nutrient partitioning [6]. In particular, volatile fatty acids produced during rumen fermentation serve as key substrates for lipid metabolism and fat deposition in muscle tissues. Consequently, forage composition, including fiber structure and fatty acid content, can influence not only animal performance but also meat quality traits such as fatty acid composition, tenderness, and flavor development [7]. Previous studies have demonstrated that forage-based feeding systems can alter both the nutritional and sensory characteristics of meat [8,9].

Goats are one of the most widely raised ruminants in Asia, where more than half of the world’s goat population is raised, owing to their adaptability to diverse environmental conditions and efficient utilization of forage resources [10]. Goat meat has attracted increasing attention due to its favorable nutritional properties, including relatively low saturated fat content and high protein levels, leading to growing production and consumption worldwide [11,12]. Although numerous studies have investigated the effects of forage feeding on growth performance, digestibility, methane emissions, and metabolic responses in goats, there remains a lack of integrated research linking forage type to comprehensive meat quality traits, including physicochemical properties, fatty acid composition, and sensory-related attributes [13,14,15].

Although previous studies have investigated the effects of forage on growth performance and nutritional responses in ruminants, direct comparisons between Bermuda grass and perennial ryegrass with respect to goat meat quality remain limited. As Bermuda grass has attracted increasing attention as a warm-season forage adapted to subtropical climates, understanding its effects on meat quality relative to the widely used perennial ryegrass is important for selecting appropriate forage resources under changing climatic conditions. Moreover, few studies have comprehensively evaluated physicochemical properties, fatty acid composition, and instrumental aroma and taste profiles simultaneously in goats. Therefore, the objective of the present study was to compare the effects of Bermuda grass and perennial ryegrass on growth performance and meat quality characteristics of goats.

## 2. Materials and Methods

### 2.1. Animals, Experimental Design, and Diets

All analyses, except for shear force determination, were performed using raw goat meat samples. In this experiment, thirty castrated Korean crossbred goats (Korean native black × Boer, 10 months old, castrated males, initial body weight: 36.1 ± 3.8 kg) were used. All goats originated from the same farm, shared the same genetic background, and were raised under identical housing, environmental, and management conditions throughout the experimental period. Both treatment groups were maintained simultaneously. A randomized complete block design was applied. The goats were first divided into two blocks according to their initial body weight (15 goats per block). Within each block, seven or eight goats were randomly assigned to the Bermuda grass or perennial ryegrass treatment, resulting in 15 goats per treatment overall. Before the feeding trial, all goats were allowed a 7-day adaptation period to acclimate to the housing conditions and experimental diets. The experimental diet consisted of 35% forage and 65% commercial concentrate (NONGHYUPFEED Co., Ltd., Seoul, Republic of Korea). The forage-to-concentrate ratio was selected to provide adequate nutrient intake during the finishing period while maintaining a practical feeding regimen for growing goats. Each goat received a fixed daily allowance of 1.15 kg of feed per day to minimize variation in nutrient intake among animals and to allow a controlled comparison of the effects of forage type on growth performance and meat quality. The goats were fed twice daily and had free access to fresh drinking water throughout the experimental period. All animals were maintained under identical housing, environmental, and management conditions. The remaining feed in each treatment group was measured daily to estimate average daily feed intake. The goats were fed the experimental diets for 4 months during the finishing period. To estimate body weight and average daily gain (ADG), the goats were weighed at 1, 2, 3, and 4 months. All animal handling and slaughter procedures were performed in accordance with the guidelines approved by the Institutional Animal Care and Use Committee (IACUC) of Sangji University (approval no. 2024-6). Following electrical stunning and slaughter at a commercial slaughterhouse, the carcasses were chilled at 4 °C for 24 h. After the completion of rigor mortis, the rectus femoris muscles were excised from each carcass, vacuum-packed, stored at 4 °C, and analyzed according to the respective experimental procedures described below.

### 2.2. Feed Chemical Composition Analysis

The nutritional composition of the experimental diets was analyzed according to the official methods of the Association of Official Analytical Chemists (AOAC) [16]. All feed composition analyses were performed in triplicate, and the reported values represent the mean of three independent measurements. Dry matter content was determined by overnight drying of ground feed samples in a vacuum oven at 100 °C (method 930.15 [16]). For crude protein analysis, a Kjeldahl analyzer (Kjeltec 2300, FOSS Analytical, Hilleroed, Denmark) was used to measure the total nitrogen content, which was converted to crude protein using a factor of 6.25 (method 984.13 [16]). Ether extract content was quantified using an extraction system (ANKOM XT15 Extractor; ANKOM Technology, Macedon, NY, USA) according to method 920.39 [16]. Neutral detergent fiber and acid detergent fiber were analyzed according to the protocols described by Van Soest et al. [17]. Ash content was determined by overnight incineration of samples at 550 °C in a muffle furnace (KMF-500, Lab Corporation, Seoul, Republic of Korea) according to method 942.05 [16]. The nutritional components of the experimental forage and commercial concentrate are listed in Table 1.

### 2.3. Meat Proximate Composition Analysis

The proximate composition of the samples was determined according to the official AOAC methods. Moisture content was analyzed using the hot air-drying method at 105 °C (AOAC 950.46 [16]), crude protein by the Kjeldahl method (AOAC 981.10 [16]), crude fat by the Soxhlet method (AOAC 920.39C [16]), and crude ash by the direct ashing method (AOAC 920.153 [16]). All proximate composition values are expressed on a dry weight basis.

### 2.4. Shear Force Analysis

Shear force was measured using a Texture Analyzer (TA1, Lloyd, Largo, FL, USA) equipped with a V-shaped blade. Prior to analysis, the raw meat samples were cooked in a chamber at 75 °C for 40 min and then cooled to room temperature. The samples were cut into cubes (1 × 1 × 1 cm; length × width × height) aligned with the muscle fibers, and cut perpendicular to the fiber direction for analysis. The test conditions were a speed of 2 mm/s, distance of 22 mm, and maximum force of 5.6 N. Shear force was expressed in Newtons (N).

### 2.5. Fatty Acid Composition Analysis

The fatty acid composition was analyzed according to the method described by Folch et al. [18]. Ground raw meat samples 20 g sample was mixed with a chloroform–methanol solution (2:1, *v*/*v*) and homogenized at 13,500 rpm for 1 min using a homogenizer (AM-5, Nihonseiki Kaisha, Tokyo, Japan). After homogenization, 20 mL of 0.88% KCl solution was added, and the mixture was centrifuged at 3000 rpm for 10 min at 2 °C using a centrifuge (Supra R22, Hanil Science, Gimpo, Republic of Korea). The upper phase was discarded, and the lower phase was filtered through Whatman No. 1 filter paper (GE Healthcare, Chicago, IL, USA). The chloroform solvent was completely evaporated under a stream of nitrogen at 35 °C for 1 h using a nitrogen concentrator (MGS-2200, Eyela Tokyo Rikakikai, Tokyo, Japan) before methylation. The concentrated lipid extract was methylated using 0.5 N NaOH in methanol and 14% boron trifluoride in methanol. The mixture was heated at 80 °C for 1 h in a water bath (JSWB-30T, JSR, Seongnam, Republic of Korea), cooled for 10 min, mixed with 2 mL of 14% boron trifluoride, and reheated under the same conditions. After cooling, 5 mL of distilled water and 2 mL of hexane were added, followed by centrifugation at 3000× *g* for 10 min at 2 °C. A 1 μL portion of the upper layer was injected into a gas chromatograph (GC; CP-8400; Varian Inc., Palo Alto, CA, USA) equipped with an HP-Innowax capillary column (100 m × 0.32 mm i.d. × 0.25 μm film thickness; Agilent Technologies, Palo Alto, CA, USA). The GC conditions were as follows: injector temperature, 225 °C; split ratio, 1:10; and carrier gas flow rate, 1 mL/min. The oven temperature program was: hold at 150 °C for 1 min, increase to 200 °C at 15 °C/min, then to 250 °C at 2 °C/min, and hold at 250 °C for 10 min. The flame ionization detector temperature was set at 280 °C. Each fatty acid was identified by comparing its retention time with that of a standard (47015-U, PUFA No. 2, Animal Source, Supelco, Bellefonte, PA, USA), and the results were expressed as percentages of the total peak area.

### 2.6. Aromatic Profile Analysis

The aroma profiling of the samples was performed using a Heracles II electronic nose (Alpha MOS, Toulouse, France). For analysis, 5 g of ground raw meat was placed in a sealed 20 mL vial and incubated at 60 °C for 20 min. Subsequently, 2.5 mL of the headspace gas was injected into the instrument. Other analytical conditions were as follows: flow rate, 250 mL/min; acquisition time, 120 s; and trap temperature, 40 °C/240 °C (desorption). The sensor response was calculated as the relative change in resistance (ΔRgas/Rair) between the resistance in the presence of volatile compounds (Rgas) and clean air (Rair). The sensor response data obtained from all individual raw meat samples (*n* = 15 per treatment) were processed using AlphaSoft software (version 2023-7.3.0; Alpha MOS, Toulouse, France) for principal component analysis (PCA) and peak intensity to visualize differences in aroma profiles between the treatment groups. The percentages shown for PC1 and PC2 represent the proportion of the total variance explained by each principal component. The discrimination index (DI) provided by the AlphaSoft software was used to evaluate the degree of separation between treatment groups, with higher values indicating better discrimination. In the PCA plot, each polygon represents the distribution area of the individual samples within each treatment group. All aroma analyses were conducted using raw meat samples without thermal processing.

### 2.7. Taste Profile Analysis

Taste profiling of the samples was performed using an Astree 5 electronic tongue (Alpha MOS, Toulouse, France). For each sample, 1 g of ground raw meat was weighed into a 50 mL conical tube, mixed with 50 mL of distilled water (18.2 MΩ), and homogenized at 10,000 rpm for 1 min using a homogenizer (AM-5, Nissei, Nagano, Japan). The homogenate was filtered through Whatman No. 1 filter paper (Whatman, Maidstone, UK), and the supernatant was collected. The extract was diluted with distilled water at a 1:1000 (*v*/*v*) ratio and placed in a 150 mL tall beaker for analysis. The taste sensors used included sourness (AHS), saltiness (CTS), and umami (NMS) sensors, along with general-purpose sensors (PKS, CPS, ANS, and SCS). The response values obtained from all taste sensors (AHS, CTS, NMS, PKS, CPS, ANS, and SCS) for all individual raw meat samples (*n* = 15 per treatment) were analyzed using AlphaSoft software (Alpha MOS, Toulouse, France). Principal component analysis (PCA) was performed to visualize differences in taste profiles between the treatment groups, and the ranking score function of the AlphaSoft software was used to compare the overall taste characteristics of the samples. The ranking score generated by the AlphaSoft software is a relative index based on the responses of the taste sensors and was used to compare the overall taste characteristics among the treatment groups. Higher ranking scores indicate stronger relative responses for the corresponding taste attribute. The discrimination index (DI) provided by the AlphaSoft software was used to evaluate the degree of separation between treatment groups, with higher values indicating better discrimination. In the PCA plot, each polygon represents the distribution area of the individual samples within each treatment group. All taste analyses were conducted using raw meat samples without thermal processing.

### 2.8. Statistical Analysis

All experimental data were analyzed using SAS software (version 9.4; SAS Institute Inc., Cary, NC, USA). The experiment was conducted using a randomized complete block design (RCBD), in which forage type (Bermuda grass or perennial ryegrass) was treated as the fixed effect and initial body weight block was included as the blocking factor. Individual goats were considered the experimental units. Growth performance data were analyzed using a two-way ANOVA including forage treatment, block, and their interaction. Meat quality parameters were analyzed using the general linear model (GLM), and block × treatment interactions were tested. Data are expressed as mean ± standard deviation (SD), and statistical significance was declared at *p* < 0.05. All analytical measurements were performed in triplicate, and the mean value of the triplicate measurements for each individual animal was used for subsequent statistical analyses.

## 3. Results

### 3.1. Growth Performance

The growth performance of goats during the fattening stage was not affected by the experimental diets (Table 2). No significant treatment × block interactions were detected for any growth performance variable (*p* > 0.05). Therefore, only the main effects of forage treatment are presented. No significant forage treatment effects were observed for any growth performance variable (*p* > 0.05).

### 3.2. Proximate Composition and Shear Force

Table 3 shows the proximate composition and shear force of the meat of goats fed different types of forage. The protein content of the Bermuda grass group was significantly lower than that of the perennial ryegrass group (*p* < 0.05), whereas the fat content was significantly higher (*p* < 0.05). And the shear force of the Bermuda grass group was significantly lower than that of the perennial ryegrass group (*p* < 0.05).

### 3.3. Fatty Acid Composition

Table 4 presents the relative fatty acid composition of goat meat from animals fed different forage types. Bermuda grass feeding resulted in significantly higher relative proportions of myristic acid (C14:0), palmitic acid (C16:0), stearic acid (C18:0), vaccenic acid (C18:1n7), eicosapentaenoic acid (C20:5n3), adrenic acid (C22:4n6), SFA, UFA, MUFA, UFA/SFA, and MUFA/SFA than perennial ryegrass feeding (*p* < 0.05). In contrast, perennial ryegrass feeding resulted in significantly higher relative proportions of oleic acid (C18:1n9), linoleic acid (C18:2n6), α-linolenic acid (C18:3n3), and the n6/n3 ratio (*p* < 0.05). No significant differences were observed for PUFA, PUFA/SFA, or several other fatty acids between the two treatment groups (*p* > 0.05).

### 3.4. Aromatic Profile

**Figure 1:**
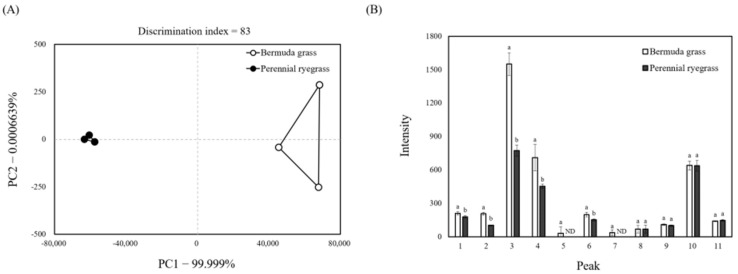
Aroma profile of the meat of goats fed different types of forage. (**A**) Principal component analysis (PCA) plot. (**B**) Volatile intensity. PC1, primary component value; PC2, secondary component value. Peaks are reported in order of elution: 1, methanol; 2, methanethiol; 3, propenal; 4, propan-2-one; 5, 3-methylfuran; 6, methyl pentanoate; 7, 2-methylheptanal; 8, 4-hydroxy-2,5-dimethyl-3(2H)-furanone; 9, citronellal; 10, methyl eugenol; 11, bis(2-methyl-3-furanyl) disulfide. Values are presented as mean ± standard deviation. ND, not detected; ^a,b^ Means in the same bars with different superscript letters differ significantly (*p* < 0.05).

Figure 1 shows the aroma profiles of the meat of goats fed the different types of forage. As shown in Figure 1A, the PCA plot clearly separated the Bermuda grass and perennial ryegrass groups along PC1 (explained variance = 99.999%). As shown in Figure 1B, the relative intensities of several volatile compounds differed significantly between the two groups (*p* < 0.05), including methanethiol (peak 2), propenal (peak 3), propan-2-one (peak 4), 3-methylfuran (peak 5), methyl pentanoate (peak 6), citronellal (peak 9), methyl eugenol (peak 10), and bis(2-methyl-3-furanyl) disulfide (peak 11).

### 3.5. Taste Profile

**Figure 2:**
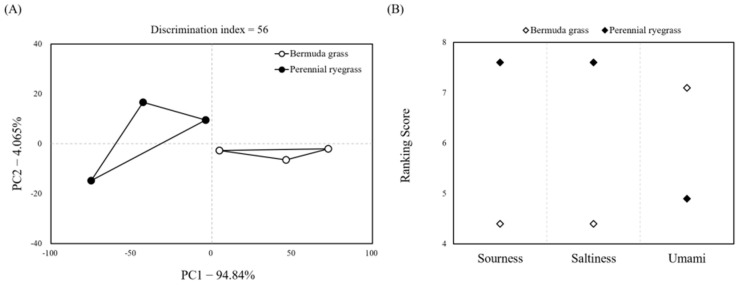
Taste profile of the meat of goats fed different types of forage. (**A**) Principal component analysis (PCA) plot. (**B**) Taste intensity. PC1, primary component value; PC2, secondary component value.

Figure 2 shows the taste profiles of the meat of goats fed different types of forage. As shown in Figure 2A, the PCA plot revealed a clear separation between the Bermuda grass and perennial ryegrass groups along PC1 (explained variance = 94.84%). The Bermuda grass group exhibited lower sourness and saltiness but a stronger umami intensity than the perennial ryegrass group (Figure 2B).

## 4. Discussion

### 4.1. Growth Performance

Although Bermuda grass contained a higher crude protein concentration (10.9%) than perennial ryegrass (4.4%), no significant differences in growth performance were observed between the treatment groups. Previous studies have suggested that differences in fiber digestibility and rumen fermentation dynamics may influence nutrient utilization and energy availability [19,20]. However, such effects were not reflected in growth performance under the experimental conditions of the present study. Although all goats underwent a 7-day adaptation period before the feeding trial, a longer adaptation period could better reflect potential differences in nutrient utilization associated with the forage sources [6], although this possibility was not directly evaluated in the present study. Therefore, further studies are needed to clarify the effects of forage source on nutrient utilization and growth performance.

### 4.2. Proximate Composition and Shear Force

West et al. [21] similarly reported that feeding Bermuda grass to Holstein and Jersey cattle increased the meat fat content, which is consistent with the results of the present study. This may be associated with differences in the nutritional composition of the forage, as variations in dietary fiber content may influence feed energy utilization efficiency [22]. Previous studies have suggested that the consumption of forage with higher dietary fiber content can enhance the rumen fibrolytic bacterial population, thereby increasing the production of volatile fatty acids such as acetate and propionate [19]. In particular, increased propionate production has been reported to influence satiety, feeding behavior, and dry matter intake, which may ultimately affect fat deposition [20]. However, because the present study evaluated only the proximate composition of the forage, the specific contribution of crude protein quality and other nutritional components could not be determined. Additional analyses, such as amino acid profiling, would provide a more comprehensive evaluation of the nutritional characteristics of the forage and help distinguish the respective contributions of forage type and nutrient composition. Therefore, the present findings should be interpreted with caution.

The lower shear force observed in the Bermuda grass group may be associated with its higher fat content. Gawat et al. [23] reported similar findings in goat meat quality, noting that increased fat content was associated with reduced shear force. Shear force, which reflects the ease of mastication, is widely used as an instrumental indicator of meat tenderness and is considered an important determinant of meat quality [24]. Owing to the negative correlation between fat content and shear force, increasing the intramuscular fat content has been considered an important objective of meat quality improvement strategies in animal breeding [25]. Therefore, these findings suggest that Bermuda grass feeding may contribute to improved tenderness through its association with higher fat content and lower shear force. However, additional sensory evaluation is warranted to determine whether these instrumental differences translate into perceptible improvements in eating quality.

### 4.3. Fatty Acid Composition

In particular, linoleic acid (C18:2n6) and α-linolenic acid (C18:3n3) are essential fatty acids that cannot be synthesized endogenously and are considered beneficial components of the human diet [26]. In the present study, the perennial ryegrass group showed significantly higher relative proportions of these essential fatty acids than the Bermuda grass group. These differences may be associated with variations in forage composition, which can influence the transfer and metabolism of dietary fatty acids in ruminants [27]. Moreover, fatty acid ratios influence indices such as the thrombogenic index, health-promoting index, and blood cholesterol levels, which are widely used to evaluate the nutritional quality of dietary lipids and their implications for cardiovascular health [28]. In the present study, Bermuda grass feeding resulted in significantly higher UFA/SFA and MUFA/SFA ratios than perennial ryegrass feeding, whereas the PUFA/SFA ratio did not differ significantly between treatments. These findings indicate that Bermuda grass and perennial ryegrass differentially influence the nutritional quality of goat meat, with Bermuda grass improving the UFA/SFA and MUFA/SFA ratios and perennial ryegrass increasing the relative proportions of essential polyunsaturated fatty acids. These findings should also be interpreted in the context of ruminant lipid metabolism. In ruminants, dietary unsaturated fatty acids undergo extensive microbial biohydrogenation in the rumen, resulting in alterations to the fatty acid composition of meat [29]. Therefore, differences in the fatty acid profiles observed between forage treatments are likely associated not only with the intrinsic fatty acid composition of the forages but also with differences in ruminal lipid metabolism. However, because the fatty acid data were expressed as relative percentages rather than absolute quantities, it remains unclear whether the observed differences reflect true changes in fatty acid abundance or relative shifts resulting from differences in the proportions of other chromatographic peaks. Therefore, these findings should be interpreted with caution until quantitative analyses using appropriate standards are performed. Overall, the present findings demonstrate that forage type differentially influences the relative fatty acid composition of goat meat. Perennial ryegrass was associated with higher relative proportions of essential fatty acids, including linoleic acid and α-linolenic acid, whereas Bermuda grass was associated with higher UFA/SFA and MUFA/SFA ratios, which may contribute to a more favorable lipid nutritional profile. Therefore, the nutritional implications of forage type should be interpreted based on the overall fatty acid profile rather than individual fatty acids alone. Future studies should focus on developing feeding strategies that simultaneously enhance essential polyunsaturated fatty acids while maintaining favorable fatty acid ratios, thereby improving the nutritional quality of goat meat.

### 4.4. Aromatic Profile

Methanethiol has been associated with creamy and savory aroma notes, whereas 2-methylheptanal has been associated with fresh and pungent aroma characteristics [30]. Propenal and propan-2-one have been reported to exhibit sweet and fruity aroma descriptors reminiscent of cherry, apple, and pear [31]. Although 3-methylfuran is generally considered odorless, methyl pentanoate has been associated with fruity aroma notes reminiscent of red wine and blackberries [32]. In the present study, differences in the relative abundance of these volatile compounds were observed between the Bermuda grass and perennial ryegrass groups, suggesting differences in volatile compound profiles rather than directly indicating differences in perceived aroma. Melton et al. [33] reported that beef from pasture-fed cattle exhibited more intense creamy and dairy-like flavor characteristics, which may be related to differences in volatile composition. Similarly, variations in fatty acid composition have been reported to influence the formation of volatile compounds, including aldehydes and esters, through lipid oxidation pathways [34]. These compounds have frequently been associated with sweet, fruity, and mildly creamy aroma descriptors. Therefore, the differences in volatile compound profiles observed in the present study may be associated with differences in fatty acid composition between the forage treatments. In particular, the higher relative proportions of linoleic acid and α-linolenic acid observed in the Bermuda grass group may influence the formation of aldehydes and esters through lipid oxidation, although this mechanism was not directly evaluated in the present study. Overall, the present findings suggest that forage type may influence the volatile compound profile of goat meat; however, whether these instrumental differences correspond to perceptible sensory differences remains unknown. Further validation using trained sensory panels is, therefore, required. In addition, because odor threshold values were not determined in the present study, it remains unclear whether the detected volatile compounds were present at concentrations sufficient to contribute to the perceived aroma of raw goat meat. Furthermore, the volatile compounds detected in raw meat may not directly reflect the aroma characteristics of cooked meat because thermal processing generates additional aroma-active compounds through lipid oxidation and Maillard reactions. Therefore, caution is required when extrapolating the present findings to cooked meat aroma.

### 4.5. Taste Profile

Umami, a savory taste, enhances flavor complexity and palatability and plays an important role in appetite stimulation [35]. Unlike sourness and saltiness, which are detected through ion channels, umami is perceived by specific taste receptors in the tongue and palatal epithelium and is particularly effective in improving palatability in low-sodium foods [36]. Differences in the electronic tongue response for the umami sensor were observed between the treatment groups. However, the compounds directly responsible for umami taste, such as free amino acids (e.g., glutamic acid and aspartic acid) and nucleotides, were not measured in the present study. Therefore, the mechanisms underlying the observed differences in electronic tongue responses remain unclear. However, the compounds directly responsible for umami taste, such as free amino acids (e.g., glutamic acid and aspartic acid) and nucleotides, were not measured in the present study. Therefore, the mechanisms underlying the observed differences in electronic tongue responses remain unclear. As the taste analysis was conducted using aqueous extracts of raw meat, the results reflect water-soluble taste compounds influenced by the dietary treatments with minimal influence from heat-induced reactions. Further studies using cooked meat would be valuable for evaluating taste characteristics under actual consumption conditions. It should be noted that the present findings are based on instrumental analyses using an electronic tongue and were not validated by trained sensory panel evaluation. Although the electronic tongue provides objective and reproducible measurements of taste-related characteristics, it cannot fully replace human sensory assessment. Therefore, future studies incorporating trained sensory panels are warranted to validate the instrumental findings obtained in the present study under practical consumption conditions.

## 5. Conclusions

Feeding Bermuda grass influenced the physicochemical and sensory-related characteristics of goat meat without affecting overall growth performance during the fattening period. The Bermuda grass group showed higher intramuscular fat content and lower shear force, indicating improved tenderness. In addition, essential fatty acids such as linoleic acid and α-linolenic acid were increased, and the taste profile was characterized by enhanced umami intensity and reduced sourness and saltiness. These changes suggest an improvement in palatability-related traits. However, the lower UFA/SFA ratio observed in the Bermuda grass group indicates a potential nutritional limitation. Therefore, although Bermuda grass feeding can enhance eating quality, further nutritional strategies are required to improve the fatty acid health index. Overall, this study provides useful information on the effects of forage type on the quality characteristics of goat meat under changing climatic conditions. Future studies should include instrumental color measurements, trained sensory panel evaluation, and multivariate statistical approaches using individual-level datasets to provide a more comprehensive assessment of the effects of forage type on goat meat quality. In addition, further research should focus on developing feeding strategies that preserve the beneficial increase in essential fatty acids while minimizing saturated fatty acid accumulation to improve the overall nutritional quality of goat meat.

## Figures and Tables

**Table 1:** Nutritional composition of experimental forage and concentrate (% of dry matter, DM).

Items	Bermuda Grass	Perennial Ryegrass	Concentrate
DM, % as-fed	93.7 ± 0.1	92.1 ± 0.1	89.1 ± 0.1
CP	10.9 ± 0.2	4.4 ± 0.1	18.3 ± 0.1
N	1.7 ± 0.1	0.7 ± 0.1	2.9 ± 0.1
EE	1.4 ± 0.1	1.0 ± 0.1	4.6 ± 0.1
NDF	71.6 ± 3.1	66.1 ± 1.8	31.4 ± 2.2
ADF	32.3 ± 2.0	36.5 ± 2.6	11.7 ± 1.0
Ash	7.3 ± 0.1	5.5 ± 0.1	8.3 ± 0.1
NFC	8.9 ± 3.4	23.1 ± 1.8	37.5 ± 2.1

Values are expressed as mean ± standard deviation. DM, dry matter; CP, crude protein; N, nitrogen; EE, ether extract; NDF, neutral detergent fiber; ADF, acid detergent fiber; NFC, non-fiber carbohydrate. Non-fiber carbohydrate (%) = 100 (CP + EE + NDF + Ash).

**Table 2:** Growth performance of goats fed diets with different forage during the fattening stages.

Items	Bermuda Grass	Perennial Ryegrass	*p*-Value		
			Treatment	Block	Block × Treatment
Initial BW (kg)	36.05 ± 3.54	36.04 ± 4.17	0.978	0.480	0.228
BW at 1 month (kg)	39.64 ± 3.03	39.39 ± 5.18	0.770	0.312	0.330
BW at 2 months (kg)	44.30 ± 2.59	43.80 ± 5.77	0.533	0.095	0.400
BW at 3 months (kg)	47.15 ± 2.60	46.91 ± 6.38	0.641	0.012	0.385
Final BW (kg)	51.13 ± 3.64	49.65 ± 7.94	0.249	0.001	0.160
ADG (g)	125.61 ± 29.41	113.44 ± 45.39	0.088	<0.001	0.245

Values are expressed as mean ± standard deviation. *p*-values for forage treatment, body weight block, and forage treatment × block interactions were obtained using two-way ANOVA. BW, body weight; ADG, average daily gain.

**Table 3:** Proximate composition and shear force of the meat of goats fed different types of forage.

Items	Bermuda Grass	Perennial Ryegrass	*p*-Value		
			Treatment	Block	Block × Treatment
Moisture content (%)	71.67 ± 0.26	71.14 ± 0.50	0.014	0.112	0.520
Protein content (%)	20.23 ± 0.05 ^b^	21.03 ± 0.10 ^a^	<0.001	0.447	0.501
Fat content (%)	7.65 ± 0.27 ^a^	6.39 ± 0.45 ^b^	<0.001	0.402	0.880
Ash content (%)	1.40 ± 0.08	1.50 ± 0.06	0.008	0.016	0.624
Shear force (N)	28.13 ± 0.80 ^b^	30.72 ± 0.81 ^a^	0.001	0.691	0.307

Values are expressed as mean ± standard deviation. *p*-values for forage treatment, body weight block, and forage treatment × block interactions were obtained using two-way ANOVA. ^a,b^ Means in the same row with different superscript letters differ significantly. All proximate composition values are expressed on a fresh weight basis.

**Table 4:** Fatty acid composition of the meat of goats fed different types of forage.

Items	Bermuda Grass	Perennial Ryegrass	*p*-Value		
			Treatment	Block	Block × Treatment
Myristic acid (C14:0)	4.51 ± 0.14 ^a^	4.13 ± 0.14 ^b^	0.003	0.488	0.702
Palmitic acid (C16:0)	40.29 ± 0.06 ^a^	39.57 ± 0.40 ^b^	0.004	0.436	0.456
Palmitoleic acid (C16:1n7)	1.55 ± 0.01	1.53 ± 0.03	0.135	0.583	0.693
Stearic acid (C18:0)	19.58 ± 0.08 ^a^	19.28 ± 0.22 ^b^	0.027	0.403	0.502
Oleic acid (C18:1n9)	27.88 ± 0.09 ^b^	29.06 ± 0.27 ^a^	<0.001	0.450	0.507
Vaccenic acid (C18:1n7)	0.88 ± 0.00 ^b^	0.95 ± 0.04 ^a^	0.012	0.762	0.830
Linoleic acid (C18:2n6)	3.72 ± 0.01 ^b^	3.81 ± 0.03 ^a^	0.001	0.461	0.419
γ-Linolenic acid (C18:3n6)	0.03 ± 0.00	0.03 ± 0.00	0.677	0.761	0.835
α-Linolenic acid (C18:3n3)	0.32 ± 0.00 ^b^	0.39 ± 0.00 ^a^	<0.001	0.402	0.519
Arachidonic acid (C20:4n6)	1.00 ± 0.01	1.02 ± 0.02	0.140	0.568	0.782
Eicosapentaenoic acid (C20:5n3)	0.05 ± 0.00 ^b^	0.06 ± 0.00 ^a^	0.001	0.468	0.813
Adrenic acid (C22:4n6)	0.12 ± 0.00 ^a^	0.11 ± 0.00 ^b^	0.017	0.647	0.430
Docosahexaenoic acid (C22:6n3)	0.01 ± 0.00	0.01 ± 0.00	0.641	0.883	0.913
Gondoic acid (C20:1n9)	0.06 ± 0.01	0.06 ± 0.01	0.367	0.756	0.942
SFA	63.29 ± 0.82 ^b^	64.35 ± 0.10 ^a^	0.037	0.673	0.529
UFA	36.71 ± 0.82 ^a^	35.65 ± 0.10 ^b^	0.037	0.673	0.529
MUFA	31.34 ± 0.67 ^a^	30.40 ± 0.08 ^b^	0.027	0.668	0.532
PUFA	5.43 ± 0.15	5.31 ± 0.03	0.177	0.718	0.495
n3	0.45 ± 0.01 ^a^	0.38 ± 0.01 ^b^	<0.001	0.896	0.418
n6	4.92 ± 0.13	4.87 ± 0.02	0.567	0.678	0.529
UFA/SFA	0.58 ± 0.02 ^a^	0.55 ± 0.00 ^b^	0.036	0.676	0.534
MUFA/SFA	0.50 ± 0.02 ^a^	0.47 ± 0.00 ^b^	0.029	0.673	0.537
PUFA/SFA	0.09 ± 0.00	0.08 ± 0.00	0.105	0.707	0.511
n6/n3	10.93 ± 0.09 ^b^	12.95 ± 0.19 ^a^	<0.001	0.522	0.391

Values are expressed as mean ± standard deviation. *p*-values for forage treatment, body weight block, and forage treatment × block interactions were obtained using two-way ANOVA. ^a,b^ Means in the same row with different superscript letters differ significantly. SFA, saturated fatty acid; UFA, unsaturated fatty acid; MUFA, monounsaturated fatty acid; PUFA, polyunsaturated fatty acid; n3: omega-3; n6: omega-6. Note: Fatty acid composition is expressed as the relative percentage of each identified fatty acid based on the total peak area of the identified fatty acids detected by GC analysis.

## Data Availability

All data generated or analyzed during this study are included in this published article.
